# Rapamycin-induced autophagy activity promotes bone fracture healing in rats

**DOI:** 10.3892/etm.2015.2660

**Published:** 2015-07-27

**Authors:** GE YANG, XUNHONG DUAN, DASHENG LIN, TEN LI, DEQING LUO, LEI WANG, KEJIAN LIAN

**Affiliations:** Department of Orthopedic Surgery, The Affiliated Southeast Hospital of Xiamen University, Orthopedic Center of People's Liberation Army, Zhangzhou, Fujian 363000, P.R. China

**Keywords:** autophagy, rapamycin, bone fracture healing

## Abstract

Autophagy is a crucial mediating process for normal bone cell function and metabolism in physiology or pathology. Rapamycin has been demonstrated to induce the autophagy pathway by inhibiting the mammalian target of rapamycin (mTOR) pathway. However, the contribution of autophagy in orthopedic diseases is rarely reported. The aim of the present study was to evaluate the capacity of pharmacologically induced autophagy to modify disease function in a rat model of bone fracture. A femur fracture model was established via surgery in adult male Sprague-Dawley rats. Rapamycin (n=63 rats) or dimethyl sulfoxide (DMSO) vehicle control (n=63 rats) was administered intraperitoneally for 2, 4 and 6 weeks, and 21 randomly selected rats were sacrificed in each group at each time point. X-ray micro-computed tomography and hematoxylin and eosin staining were used to evaluate the extent of fracture healing in each group. The effects of rapamycin on autophagy, mTOR signaling and the expression levels of vascular endothelial growth factor (VEGF) and proliferating cell nuclear antigen (PCNA) were analyzed using immunohistochemistry, immunofluorescence staining and western blot analysis. Rapamycin affected the mTOR signaling pathway in rats following fracture, as indicated by the inhibition of the phosphorylation of ribosomal protein S6, a target of mTOR, and activation of microtubule-associated protein 2 light chain 3, a key marker of autophagy. Histomorphometry and image examination indicated that the number of osteoblasts in each section was significantly (P<0.01) increased in the rapamycin group compared with the control group, and this was associated with a significant (P<0.05) increase in mineralized callus fraction. Furthermore, rapamycin treatment increased the expression levels of VEGF and PCNA in the rat callus tissue. These results suggest that rapamycin may serve a beneficial function in fracture healing, and that the underlying mechanism may involve the activation of autophagy.

## Introduction

Bone fracture is a common injury, which may initiate a series of biophysiological and pathological reactions. These reactions may lead to fracture healing, but may additionally result in tissue damage. A number of promising therapeutic approaches have been developed, such as improvement of internal fixation devices and the application of novel biological materials; however, delayed healing or nonunion may occur in 5–10% of fractures, adding further to patient morbidity and the expense of treatment (1). The improvement of patient morbidity and reduction of costs is an incentive for the development of novel therapies to enhance fracture healing (2).

Autophagy is the process of cellular ‘self-digestion’, and serves an essential role in energy and nutrient regulation, in addition to the removal of damaged and dysfunctional macromolecules and organelles (3,4). At the cellular level, failure of autophagy results in the increased expression of abnormal genes, and may lead to cell death (5). Potential consequences of autophagy failure at the tissue and organismal level include neurodegeneration, cardiomyopathies, abnormal skeletal development and premature mortality (6–8). Microtubule-associated protein 2 light chain 3 (LC3-II) is considered to be a primary marker of autophagy (9).

The mammalian target of rapamycin (mTOR) complex is a crucial suppressor of autophagy, functioning upstream of the autophagy-related proteins, and is centrally regulated by multiple upstream signaling pathways involving phosphatidylinositol-3-kinase (PI-3K)/Akt and adenosine monophosphate-activated protein kinase (10). Furthermore, imbalances in the mTOR pathway are involved in cardiac hypertrophy, inflammatory diseases, and diabetes, and pharmacological intervention of mTOR has been proposed as a potential treatment for these conditions (11). Rapamycin is a lipophilic macrolide antibiotic that is used as an immunosuppressive drug in solid organ transplantation, and is able to induce autophagy by inhibiting ribosomal protein S6 (rpS6), a downstream target of mTOR complex 1 (mTORC1) phosphorylation (12,13). In addition, rapamycin treatment has been demonstrated to extend lifespan in mice (14), and protects against aging-associated pathologies of the brain and heart (15–18). However, to the best of our knowledge no prior studies have investigated the effects of autophagy on bone fracture healing following an intervention affecting the mTOR pathway.

Bone fractures may impair cellular homeostasis and induce significant stress in bone cells, leading to the activation of the autophagy pathway. Therefore, the aim of the present study was to investigate the effects of the pharmacological enhancement of autophagy on the process of experimental fracture healing in rats.

## Materials and methods

The study was performed in accordance with protocols of the local governmental animal care committee and the Institutional Animal Care and Use Committee at Xiamen University (Zhangzhou, China). Every effort was made to minimize animal suffering and to reduce the number of animals used.

### 

#### Experimental groups and surgical procedure

A total of 126 adult male Sprague-Dawley rats weighing 250–270 g were obtained from Xiamen University. A total of 63 rats in the rapamycin group received a daily intraperitoneal injection of rapamycin (1 mg/kg body weight/dose) from the day of fracture until they were sacrificed at 2, 4 or 6 weeks after fracture. Subsequently, the degree of fracture healing was analyzed using radiological (n=15 each at 2, 4 and 6 weeks), hematoxylin and eosin (H&E) staining (n=12), western blot analysis (n=12), immunohistochemistry (n=12) and immunofluorescence (n=12) methods. Rats in the vehicle-treated control group (n=63) received 0.4% dimethyl sulfoxide (DMSO) in a total injection volume of 0.3 ml. Among these animals, fracture healing was analyzed using radiological (n=15 each at 2, 4 and 6 weeks), H&E staining (n=12 each), western blot analysis (n=12 each), immunohistochemistry (n=12 each) and immunofluorescence (n=12 each) methods. For surgery, the rats were anesthetized by intraperitoneal injection of ketamine (75 mg/kg) and xylazine (25 mg/kg), which was provided by the Affiliated Southeast Hospital of Xiamen University. The right femur of each animal was exposed and a wire saw was used to make a middle transverse fracture, which was stabilized using a 1.0-mm diameter Kirschner wire as described previously (19). The resulting fracture was of type A3.2, according to the Müller AO classification of fractures (20). X-ray imaging (Multix TOP; Siemens, Forchheim, Germany) was used to document the positions of the implants.

#### X-ray radiography and micro-computed tomography (microCT) imaging

Fractured limbs were observed at 2, 4 and 6 weeks by performing posteroanterior X-ray radiography to record callus formation. Subsequently, fractured limbs were dissected free of soft tissues, and the intramedullary Kirschner wires were extracted to facilitate scanning using a µCT 40 micro-CT device (Scanco Medical AG, Brüttisellen, Switzerland) at 2,800 views, 5 frames per view, 35 kV and 35 µA. Three-dimensional (3D) images were rendered, and the areas of the transverse section and of void spots in each transverse section image were evaluated using VGStudio MAX software (Dürr, Bietigheim-Bissingen, Germany). The fraction of mineralized callus was quantified by calculating the total void area (which indicates the degree of residual non-mineralized tissue) as a percentage of the total area of the transverse section image.

#### Histomorphometric analyses

Fractured limbs were harvested and fixed in 4% formalin for 24 h, decalcified in 10% ethylenediaminetetraacetic acid solution for 1 month, and embedded in paraffin for histological analysis. Decalcified longitudinal sections were cut to 4–5 µm, stained with H&E and the total number of osteoblasts in each callus section was counted.

#### Immunohistochemistry

Sections from paraffin-embedded samples were deparaffinized using the xylene substitute Pro-Par Clearant (Anatech, Ltd., Battle Creek, MI, USA) and rehydrated in graded ethanol and water. For antigen unmasking, sections in 10 mM sodium citrate buffer (pH 6.0) were heated in a microwave oven at 80–85°C for 2 min. Slides were then cooled at room temperature for 30 min. After washing with phosphate-buffered saline (PBS), sections were blocked with 5% serum for 30 min at room temperature. Polyclonal anti-phospho-rpS6 primary antibody (1:100; Abcam, Cambridge, UK) was applied and the sections were incubated overnight at 4°C. After washing with PBS, sections were incubated with polyclonal biotinylated goat anti-rabbit secondary antibody (1:100; Abcam) for 1 h at 37°C and incubated with Vectastain ABC-alkaline phosphatase (AP) (Vector Laboratories, Inc., Burlingame, CA, USA) for 30 min. Slides were washed 3 times with PBS, and sections were incubated with AP substrate for 30 min.

#### Immunofluorescence

Paraffin-embedded samples were deparaffinized using Pro-Par Clearant and rehydrated in graded ethanol and water. After washing with PBS, sections were blocked with 5% serum for 30 min at room temperature, then incubated with rabbit anti-LC3-II polyclonal antibody (1:150; Abcam) overnight at 4°C. After washing with PBS, sections were incubated with polyclonal Alexa Fluor 488 anti-rabbit IgG secondary antibody (1:200; Abcam) for 1 h. Finally, slides were washed and mounted with ProLong Gold Antifade Reagent (Invitrogen Life Technologies, Carlsbad, CA, USA).

#### Western blot analysis

Rats were anesthetized and the soft tissue covering the diaphyseal part of the femora was removed prior to sacrifice. The visible callus was resected and frozen in nitrogen. For extraction of the whole-protein fraction, tissue samples were homogenized in 250 µl radioimmunoprecipitation assay buffer, containing 150 mM NaCl, 1% NP-40, 25 mM Tris-HCl (pH 7.6), 0.1% sodium dodecyl sulfate polyacrylamide (SDS), 1% sodium deoxycholate and a protease inhibitor cocktail (Abcam). Next, the samples were incubated for 30 min on ice and centrifuged for 30 min at 16,000 × g. The supernatant was saved as a whole-protein fraction, and the protein concentration was by Lowry assay. Subsequently, 60 mg protein per lane was separated on 10% SDS gel and transferred to a polyvinyldifluoride membrane (Thermo Scientific, Waltham, MA, USA). After blocking with 5% skimmed milk solution for 1 h, membranes were incubated for 2 h with 0.5 µg/ml rat monoclonal anti-VEGF (Abcam) or 0.5 µg/ml anti-PCNA (Abcam) antibodies. Antibody-protein complexes were visualized using chemiluminescence (ProLong Gold Antifade Reagent) and photographs of the specific protein bands were captured with a Fusion FX7 imaging system (Vilber Lourmat Deutschland GmbH, Eberhardzell, Germany). Quantity One software (Bio-Rad Laboratories GmbH, Munich, Germany) was used for the analysis and quantification of the images. Glyceraldehyde 3-phosphate dehydrogenase was used as an internal control.

#### Statistical analysis

Results are presented as the mean ± standard deviation. Comparisons between two groups were conducted with t-tests. Statistical analysis was performed using SPSS version 20.0 (IBM, Armonk, NY, USA). P<0.05 was considered to indicate a statistically significant difference.

## Results

### 

#### Systemic administration of rapamycin modulates mTOR signaling and autophagy in a rat fracture model

**Figure 1.**
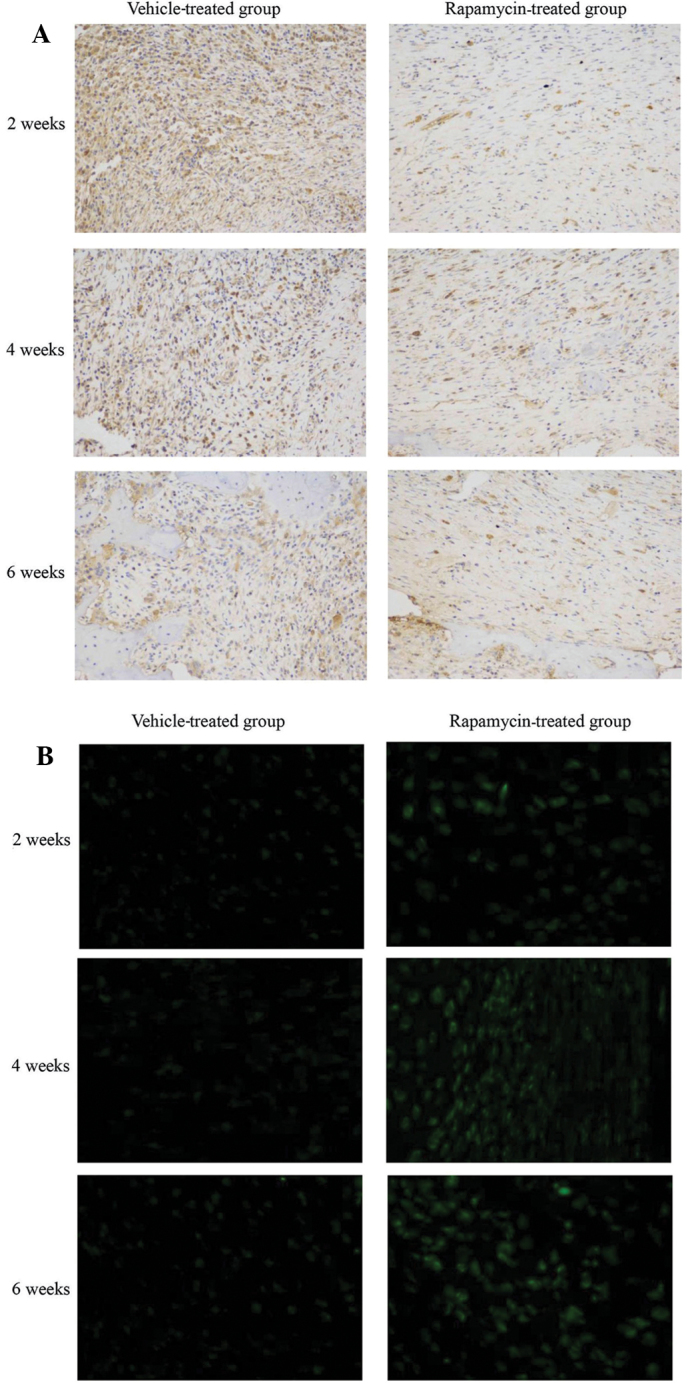
Systemic administration of rapamycin modulates the mammalian target of rapamycin signaling pathway and autophagy in a rat fracture model. Calluses from rats were collected at 2, 4 and 6 weeks post-fracture after treatment with rapamycin or the vehicle (n=12 per group). (A) Sections were analyzed using immunohistochemistry for phosphorylation of ribosomal protein S6. (B) Sections were analyzed by immunofluorescence for light chain 3-II (magnification, ×200).

The phosphorylation levels of rpS6, a downstream target of mTORC1 (21), were determined to evaluate the effect of rapamycin on the mTOR signaling pathway in a rat femur fracture model. Rapamycin treatment suppressed rpS6 phosphorylation in bone tissue cells in the callus compared with that in vehicle-treated rats (Fig. 1A). In order to determine whether autophagy is promoted as a result of mTOR inhibition by rapamycin, bone tissue sections were stained with LC3-II antibody. An increase in LC3-II expression was detected following rapamycin treatment. This increase correlated with an increase in LC3-II puncta, indicating a marked activation of autophagy in the bone tissue (Fig. 1B). These results indicate that mTOR signaling and autophagy were induced in bone tissue by the intraperitoneal administration of rapamycin.

#### Rapamycin promotes callus formation and remodeling

**Figure 2.**
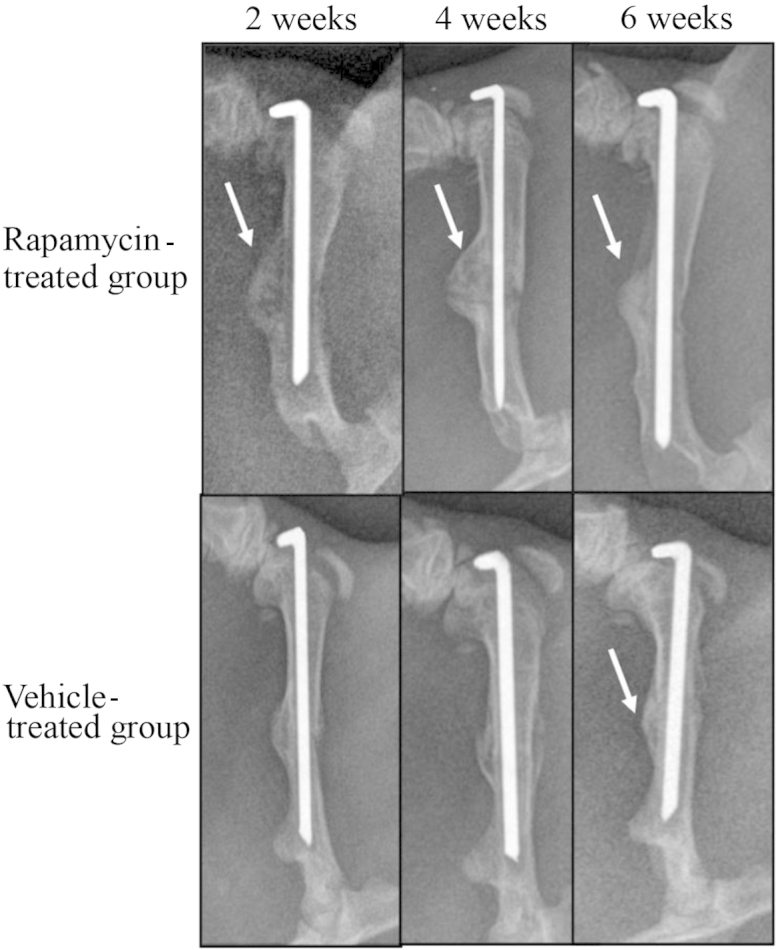
Effect of rapamycin on the size and mineralization of fracture calluses during bone repair. Representative X-ray images of fracture calluses from rapamycin and vehicle-treated rats at 2, 4 and 6 weeks post-fracture. Arrow indicates density of mineralization with fracture calluses.

To investigate the role of mTOR in fracture repair, radiographic analyses of the healing bones were conducted at 2, 4 and 6 weeks after fracture (Fig. 2). The calluses from the rats in the vehicle-treated group were not mineralized at 2 weeks after fracture. At 4 weeks, the calluses in this group exhibited a higher mineral density. At 6 weeks post-fracture, calluses from the vehicle-treated group appeared to have been resorbed, as indicated by their reduced size compared with that at week 4. In contrast with the vehicle-treated group, fracture calluses from the rapamycin-treated group were mineralized at 2 weeks post-fracture. By week 4, the calluses from the rapamycin group were increased in size compared with those from the vehicle-treated group. The calluses from the rapamycin group closely resembled those from the vehicle-treated group at 6 weeks, with resorption of the calluses; however, the mineral density was increased in the rapamycin group compared with that in the vehicle-treated group.

**Figure 3.**
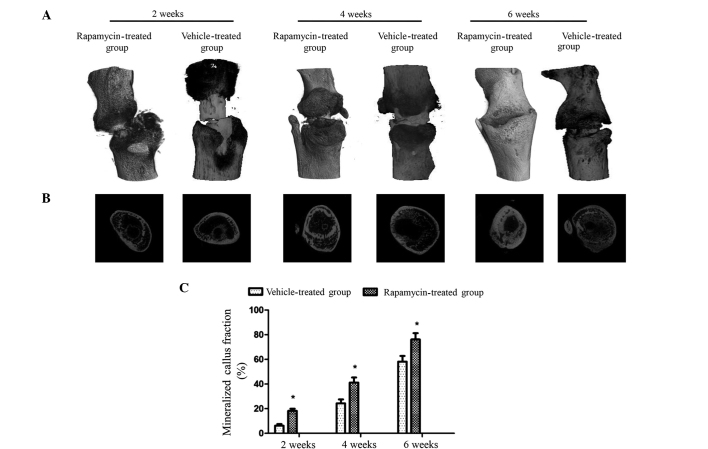
Influence of rapamycin on fracture callus mineralization and remodeling. Bone fracture global imaging was used to rebuild bone structure by micro-computed tomography (micro-CT). All fracture callus sites from rapamycin- and vehicle-treated rats were harvested at 2, 4 and 6 weeks post-fracture (n=15 per group). (A and B) Global image and transverse section of fracture calluses were scanned and graphically re-constructed using micro-CT. (C) Mineralized callus fraction was analyzed using micro-CT. Values are presented as the mean ± standard deviation. *P<0.05 vs. the vehicle-treated group.

Micro-CT imaging was used to remodel the fracture calluses and to quantify the degree of mineralization at 2, 4 and 6 weeks post-fracture (Fig. 3A). 3D representations of the fracture showed that at 2, 4 and 6 weeks post-fracture, the mineralized fracture calluses in the rapamycin group were larger than those in the vehicle-treated group. Transverse sections demonstrated that the rapamycin group exhibited mineralized fracture calluses with a higher degree of mineralization compared with those in the vehicle-treated group at all three time points (Fig. 3B). Quantitative analysis, which involved determining the total percentage area of void regions in the transverse section image indicated that the fraction of mineralized callus in the rapamycin group rats was increased with time by 18.3±1.6, 41.2±4.1 and 76.2±5.1% at 2, 4 and 6 weeks post-fracture, respectively. These values are significantly increased compared with those in the vehicle-treated group at each time point (Fig. 3C). These data indicate that treatment with rapamycin is able to enhance callus mineralization and promote callus formation and remodeling in this rat fracture model.

#### Rapamycin increases the number of osteoblasts in the rat callus, and the expression levels of PCNA and VEGF in cells

**Figure 4.**
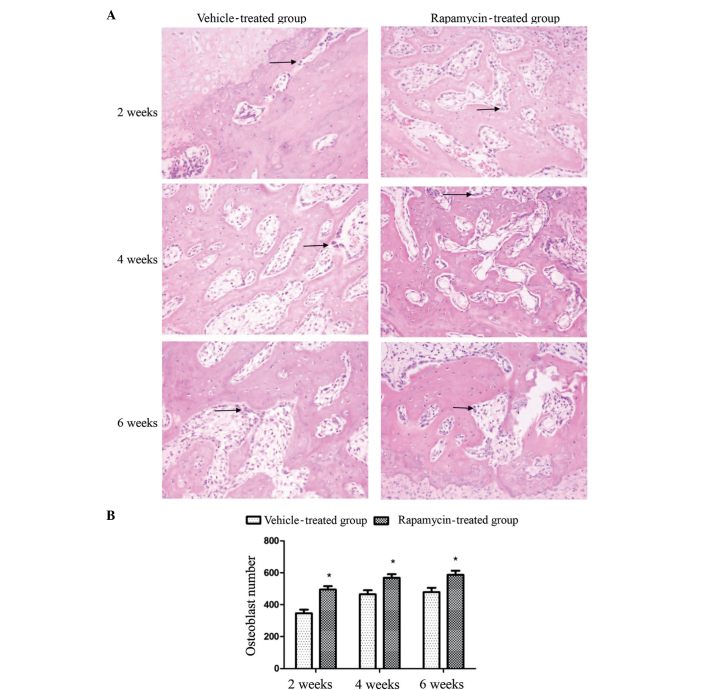
Rapamycin increase the number of osteoblasts in the rat callus. (A) Calluses section from Sprague-Dawley rats at 2, 4 and 6 weeks post-fracture under treatment with rapamycin or vehicle were stained with hematoxylin and eosin (n=12 per group; magnification, ×200). Arrow indicates an osteoblast in the callus. (B) Quantitative analysis of osteoblast number indicated a significant increase in cellularity after rapamycin treatment compared with the vehicle treatment. Values are presented as the mean ± standard deviation. *P<0.01 vs. vehicle-treated group.

To investigate the mechanism of action of rapamycin in the rats, a total of four sections were observed for each group at each time point to analyze the osteoblast cell density in the callus. It was observed that osteoblast formation occurred at a greater extent in the rats of the rapamycin group at the three time points post-fracture (Fig. 4A), and the difference in osteoblast number between the two groups was statistically significant (P<0.01; Fig. 4B).

**Figure 5.**
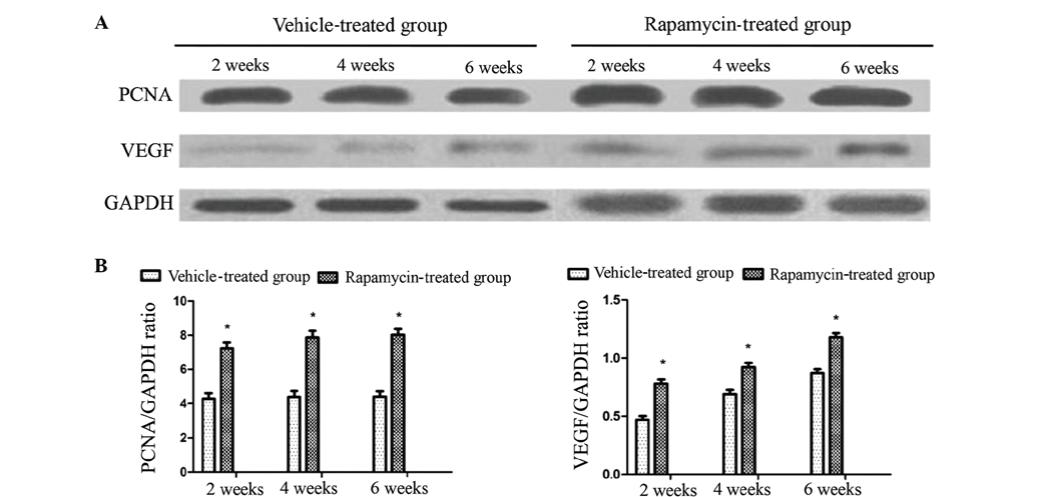
Effects of rapamycin on PCNA and VEGF expression in the callus. (A) Western blot analysis of PCNA and VEGF expression levels in the rat callus at 2, 4 and 6 weeks post-fracture. (B) Quantitative comparison of PCNA and VEGF expression levels between vehicle-treated and rapamycin groups, expressed as a ratio of GAPDH, used as loading control (n=12 per group). Values are presented as the mean ± standard deviation. *P<0.05 vs. vehicle-treated group. PCNA, proliferating cell nuclear antigen; VEGF, vascular endothelial growth factor; GAPDH, glyceraldehyde 3-phosphate dehydrogenase.

Western blot analysis was performed to determine the expression levels of PCNA and VEGF. PCNA is a crucial protein for the proliferation of osteoblasts, and VEGF is known to be a crucial molecule in the promotion of angiogenesis during fracture healing. Increased expression levels of PCNA and VEGF were detected in the rapamycin group rats compared with the vehicle-treated rats at each time point. This increase was statistically significant (P<0.05; Fig. 5). This finding indicates that rapamycin contributes to osteoblast proliferation by promoting PCNA and callus blood supply through the promotion of VEGF expression.

## Discussion

There is evidence to suggest that effective autophagy exists within a narrow homeostatic range to regulate protein homeostasis and cell survival (22). To a certain extent, autophagy affects bone formation or loss (23,24). However, to the best of our knowledge there are no previous studies regarding the effects of autophagy on bone repair. On this basis, the aim of the present study was to investigate whether the activation of autophagy affects early bone fracture healing. Rapamycin was selected as an inhibitor of the mTOR signaling pathway, which regulates the initiation of autophagy. Rapamycin is a lipophilic macrolide antibiotic that is used as an immunosuppressive drug and to induce autophagy in a variety of cell types (12–18).

The results of the present study suggest that the systemic administration of rapamycin inhibits the mTOR signaling pathway in the rat callus, as indicated by the reduced phosphorylation of rpS6, which integrates the processes of protein translation with cell growth and proliferation (25). Treatment of cells with rapamycin blocks S6 kinase 1 (S6K1) phosphorylation, and inhibits the activation of S6K1 (26). Furthermore, rapamycin-activated autophagy was indicated by increased expression levels of LC3-II, the most specific autophagosomal marker in the rat callus. These results confirm that the systemic administration of rapamycin modulates mTOR signaling and autophagy in a rat fracture model.

In the present study, treatment with rapamycin was observed to induce a significant improvement in callus formation and mineralization. In fracture healing, the fraction of mineralized callus is an important parameter, which indicates the presence of newly formed bone and the effect of bone formation or remodeling. Surface calluses formed more rapidly in the rapamycin group than in the vehicle-treated group. Furthermore, mineralization in the fracture calluses of the rapamycin group was significantly increased compared with that in the vehicle-treated group at 2, 4 and 6 weeks post-fracture.

On the basis of the established efficacy of rapamycin in the promotion of callus formation, the expression of VEGF was investigated, as a key growth factor for the promotion of endochondral ossification during secondary fracture healing. This stage of fracture healing comprises: i) Osteocyte survival and cell death; ii) degradation and calcification of the extracellular matrix; and iii) the formation of new blood vessels and new bone tissue (27). VEGF is considered to exert a direct effect on osteoprogenitor cells by promoting osteoblast differentiation and increasing the mineralization of regenerated bone (28). Therefore, the positive effect of rapamycin on fracture repair may be due to the activation of VEGF expression in callus tissue, as suggested by western blot analysis. The increased expression of VEGF may be associated with altered cell proliferation, as indicated by the elevated PCNA levels in the calluses of the rapamycin group. These results are consistent with a previous study in which rapamycin increased the cellular levels of PCNA (29).

Histological analyses showed that the number of osteoblasts in the callus at different time points differed significantly between the rapamycin and vehicle-treated group rats. This difference may be associated with the increased levels of PCNA; thus, it was hypothesized that the observed effects on fracture healing, VEGF and PCNA expression, and osteoblast activity were a result of the inhibition of mTOR by rapamycin. Rapamycin is a specific inhibitor of mTOR, particularly for the mTORC1 protein, and there is no evidence that it exerts off-target effects on enzymes other than the mTOR kinase (30,31). mTOR inhibition is known to induce autophagy; however, other signaling pathways are affected directly or indirectly by mTOR inhibition, such as the PI-3K/Akt signaling pathway (32). With regard to the role of autophagy activation in the effects observed in the present study, it is possible that rapamycin restores the suppressed autophagy, which a previous study had observed in cartilage and osteoarthritis (33), and as result, inhibits cell death. The induction of cell death is a well-known effect of defective autophagy (34). Therefore, it appears that the preservation of osteoblasts in calluses and the promotion of fracture reparation observed in the present study are attributable to the activation of autophagy.

The promotion of VEGF and PCNA expression in the rapamycin-treated rats may be associated with the increased levels of autophagy and mTORC1 inhibition. Previous studies have observed that decreased levels of autophagy and mTORC1 activation are associated with aging-related bone loss and other pathologies (35,36). Conversely, increased levels of autophagy and a reduction in mTORC1 expression may lead to an extension of lifespan (37,38). However, the increased VEGF and PCNA expression levels observed in the rapamycin-treated rats in the present study are inconsistent with those of a previous study by Holstein *et al* (39). In this study, Holstein *et al* observed that rapamycin initially delayed fracture healing and reduced VEGF and PCNA expression. It is plausible that this discrepancy is a result of the different doses of rapamycin used in the two studies, or the different animals. Further studies may be required to investigate the effect of various concentrations of rapamycin on bone healing.

There are a number of limitations to the present study. For example, the effect of rapamycin-induced autophagy on the formation of osteoclasts and osteocytes was not investigated, and the results of animal studies do not always correlate with results in humans. However, the most notable limitation of the present study is that rapamycin is a therapeutic immunosuppressant drug, which is used widely to treat cancer and reduce organ transplant rejection. The clinical feasibility of the application of mTOR inhibitors to the treatment of fractures may be enhanced by recent advances in the development of novel rapamycin analogs that exhibit improved safety. Rapamycin analogs that are more specifically targeted may activate autophagy with fewer side-effects (40).

The effects of rapamycin on fracture healing have been well documented by Holstein *et al* (41); however, these authors did not investigate the phosphorylation of rpS6 and the activation of LC3-II. To the best of our knowledge, the present study is the first to investigate the therapeutic benefits of rapamycin-induced autophagy in an animal model of fracture. Adult rats were subjected to surgical femur fracture stabilization, which is a widely used model. For further preclinical development of autophagy activators, future studies may be required to investigate the effects of autophagy on fracture healing in older animals, in order to more realistically model the human condition.

In summary, rapamycin was used to induce the activation of autophagy in rats and the efficacy of this intervention on fracture healing was investigated. These results indicate that the pharmacological inhibition of mTOR and the induction of autophagy by rapamycin may be an effective therapeutic approach for the promotion of fracture healing.
