# Rapamycin-induced autophagy activity promotes bone fracture healing in rats

**DOI:** 10.3892/etm.2021.9749

**Published:** 2021-02-03

**Authors:** Ge Yang, Xunhong Duan, Dasheng Lin, Ten Li, Deqing Luo, Lei Wang, Kejian Lian

Exp Ther Med 10:1327–1333, 2015; DOI: 10.3892/etm.2015.2660

Following the publication of the above article, an interested reader drew to the authors’ attention that, in Fig. 1B on p. 1329, there were striking similarities between the ‘2 weeks’ and ‘6 weeks’ Vehicle-treated group panels.

The authors re-examined their original data, and realized that ‘6 weeks’ Vehicle-treated group panel had been selected incorrectly for this Figure. The corrected version of Fig. 1, showing the correct data for the ‘6 weeks’ Vehicle-treated group panel in Fig. 1B, is shown opposite. Note that the correction of the data panel in this figure does not affect the overall conclusions reported in the paper. The authors are grateful to the Editor of *Experimental and Therapeutic Medicine* for allowing them the opportunity to publish this corrigendum, and apologize to the readership for any inconvenience caused.

## Figures and Tables

**Figure 1:**
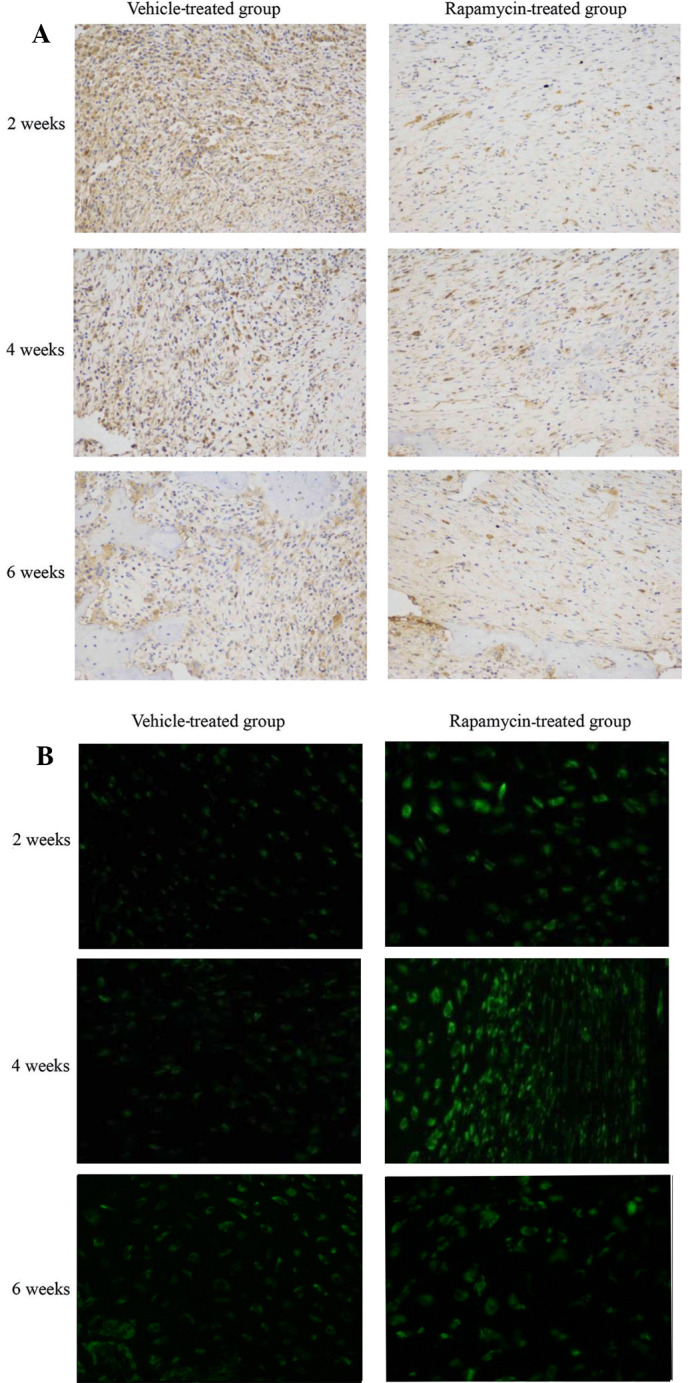
Systemic administration of rapamycin modulates the mammalian target of rapamycin signaling pathway and autophagy in a rat fracture model. Calluses from rats were collected at 2, 4 and 6 weeks post-fracture after treatment with rapamycin or the vehicle (n=12 per group). (A) Sections were analyzed using immunohistochemistry for phosphorylation of ribosomal protein S6. (B) Sections were analyzed by immunofluorescence for light chain 3-II (magnification, ×200).

